# Predicting lifespan-extending chemical compounds for *C. elegans* with machine learning and biologically interpretable features

**DOI:** 10.18632/aging.204866

**Published:** 2023-07-13

**Authors:** Caio Ribeiro, Christopher K. Farmer, João Pedro de Magalhães, Alex A. Freitas

**Affiliations:** 1 School of Computing, University of Kent, Canterbury, Kent, UK; 2 Centre for Health Services Studies, University of Kent, Canterbury, Kent, UK; 3 Genomics of Ageing and Rejuvenation Lab, Institute of Inflammation and Ageing, University of Birmingham, Birmingham, UK

**Keywords:** lifespan-extension compounds, longevity drugs, machine learning, feature selection

## Abstract

Recently, there has been a growing interest in the development of pharmacological interventions targeting ageing, as well as in the use of machine learning for analysing ageing-related data. In this work, we use machine learning methods to analyse data from DrugAge, a database of chemical compounds (including drugs) modulating lifespan in model organisms. To this end, we created four types of datasets for predicting whether or not a compound extends the lifespan of *C. elegans* (the most frequent model organism in DrugAge), using four different types of predictive biological features, based on: compound-protein interactions, interactions between compounds and proteins encoded by ageing-related genes, and two types of terms annotated for proteins targeted by the compounds, namely Gene Ontology (GO) terms and physiology terms from the WormBase’s Phenotype Ontology. To analyse these datasets, we used a combination of feature selection methods in a data pre-processing phase and the well-established random forest algorithm for learning predictive models from the selected features. In addition, we interpreted the most important features in the two best models in light of the biology of ageing. One noteworthy feature was the GO term “Glutathione metabolic process”, which plays an important role in cellular redox homeostasis and detoxification. We also predicted the most promising novel compounds for extending lifespan from a list of previously unlabelled compounds. These include nitroprusside, which is used as an antihypertensive medication. Overall, our work opens avenues for future work in employing machine learning to predict novel life-extending compounds.

## INTRODUCTION

Old age is a major risk factor for a number of diseases, including many types of cancer, cardiovascular and neurodegenerative diseases [1–3]. Hence, there has been growing interest in developing interventions that target the biological process of ageing, in order to extend lifespan and healthspan [4, 5]. Non-pharmacological interventions like dietary restriction and genetic interventions have been quite successful for extending the lifespan of model organisms [6–9]. However, genetic interventions are difficult to apply to humans, and arguably relatively few people would be willing to undergo dietary restriction in the long term. Hence, pharmacological interventions are currently the most promising type of anti-ageing intervention for extending human lifespan and healthspan, and this is current a very active research area in the biology of ageing [10–12].

A large number of compounds have been found by *in vivo* experiments to be able to prolong the lifespan of model organisms – in particular, the DrugAge database contains data on 1096 compounds that have been shown to extend the lifespan of model organisms [13]. Intuitively, the analysis of such data can lead to the discovery of novel lifespan-extending compounds, as well as potentially a further understanding of the underlying mechanisms of the biology of ageing [14, 15].

However, it is not feasible to manually analyse the relatively large volumes of data in DrugAge or other databases describing how each compound interacts with the biology of an organism. Hence, a promising research direction consists of analysing the data in such databases using machine learning algorithms that highlight patterns in data, particularly classification algorithms, which learn predictive models from data [16]. Therefore, recently there has been growing interest on applying classification algorithms to the data in DrugAge [17–20], in order to learn models that predict which compounds are more likely to extend the lifespan of a given organism, which is also the overall goal of this work.

In this context, we have prepared datasets with four different types of features describing the properties of chemical compounds (including drugs) or proteins interacting with those compounds, and trained classification models using supervised machine learning (ML) methods to predict whether or not a compound significantly extends the lifespan of *C. elegans* worms, based on data in DrugAge and other databases.

The most widely used model organism for studying biological mechanisms of ageing is *C. elegans*. It has several characteristics that facilitate *in vivo* experiments and ageing research, such as being easy and inexpensive to maintain, having a short lifespan of about 3 weeks, being easy to observe and having fully sequenced genes that are in great part homologous to human genes [21]. In addition, of particular relevance to this work, they are the model organism with the largest amount of data in the DrugAge database (667 out of 1096 entries) [13].

The computational process of knowledge discovery goes from the collection and preparation of the data to the analysis of the patterns found by the ML algorithms. During the data preparation process, for datasets with a large number of features (also called variables), like our datasets, it is common to perform a pre-processing task of feature selection (FS), which involves analysing the relationships in the data to select the most relevant features (independent variables) for the task at hand. We focused on the task of FS in this study, more specifically on filter methods, which rank features based on their relationship to the classification task, i.e., how much they influence the value of the class (target) variable. Hence, we applied filter methods in a pre-processing phase and used the selected features to train a classifier using the well-known random forest classification algorithm [22]. The most accurate predictive models were then further analysed to identify the features most relevant for our classification task and to identify novel compounds which have a high probability of extending the lifespan of *C. elegans*, as estimated by those best models.

The datasets used in our experiments were generated following a methodology which is broadly similar to the one used in the study by Barardo et al. [17]. In their study, the authors used Random Forest (RF) classifiers for the same prediction problem, also using *C. elegans* DrugAge data to obtain the instances (ageing-related compounds). That study, similarly to other related works [18, 19], uses a combination of GO terms and chemical descriptors of the compounds, applying ML techniques to the same problem of discovering compounds related to *C. elegans*’ longevity.

The novel contributions of our study compared to the related work in [17–20] are as follows. First, we created datasets using four different types of predictive features, based on Gene Ontology (GO) terms, drug (compound)-protein interactions, interactions between compounds and proteins encoded specifically by ageing-related genes, and physiology terms from a Phenotype Ontology for *C. elegans*. GO terms have been used as predictive features in [17, 18], but the other three types of features proposed here are new types of features for predicting a compound’s effect on the lifespan of an organism using machine learning, to the best of our knowledge. Note that we do not use chemical descriptors as features, an approach used in [17–20], which generated models with good predictive accuracy. We do not use chemical features because they represent very specific chemical information which is not very meaningful for biogerontologists. For instance, the three most important molecular descriptors in the best model learned in [19] were ‘number of nitrogen atoms’, ‘total positive van der Waals surface area of atoms with a partial charge in the range of 0.10 to 0.15’, and ‘hydrophobic volume’; which do not shed light on the kind of biological process associated with a drug. Hence, instead of such chemical descriptors, we use only biologically interpretable features, representing potentially relevant information for biogerontologists.

The second contribution of this study is the proposal and evaluation of an approach that automatically selects the best filter method for feature selection from a set of 5 candidate filter methods, using the training data. Finally, as additional contributions, we also perform a biological analysis of the most important predictive features in the best (most accurate) classification models learned from our datasets, and identify promising new compounds for extending *C. elegans* longevity, i.e., compounds that have not yet been associated with an increased lifespan of *C. elegans,* but are predicted to be by our best models, with a very high probability.

## Results and Discussion

As mentioned earlier, our experiments were performed on datasets created from data available in the DrugAge database and other sources (see Section 4.1). In these datasets, each instance (record) represents a compound (drug), which consists of a set of predictive features (variables) and a class label to be predicted. The class labels indicate whether or not a compound was found to significantly extend *C. elegans*’ lifespan, represented as a positive or negative class label, respectively. In essence, a compound is assigned a positive class if there is an entry in the DrugAge database [13] showing that the compound extended the average lifespan of *C. elegans* by at least 5% and the extension was statistically significant, whilst the list of negative-class compounds was obtained mainly from related work [17], consisting of compounds which do not satisfy the above criterion for the positive class (see Section 4.1 for a more precise definition of the negative class).

**Figure 1:**
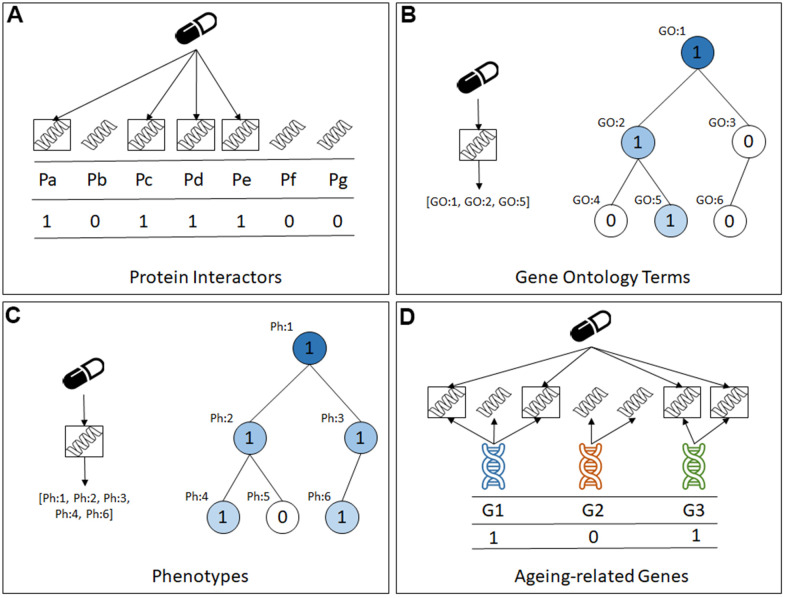
**The four types of predictive features in the datasets created for this study.** (**A**) Protein Interactors, (**B**) Gene Ontology Terms, (**C**) Phenotypes, (**D**) Ageing-related genes.

The datasets have approximately the same instances (compounds) and class labels, but each dataset has a different set of binary predictive features, as graphically summarised in Figure 1. More precisely, the four types of features (datasets) are: (a) Protein interactors: in this type of dataset each feature represents a protein and takes the value 1 or 0 to indicate whether or not that protein interacts with the compound associated with the current instance; (b) Gene Ontology (GO) term annotations: in this type of dataset each feature represents a GO term [23] and takes the value 1 or 0 to indicate whether or not the compound associated with the current instance interacts with at least one protein that is annotated with that GO term; (c) Physiology phenotype annotations: in this type of dataset each feature represents a physiology term from the WormBase Phenotype Ontology [24], and takes the value 1 or 0 to indicate whether or not the compound associated with the current instance interacts with at least one protein that is annotated with that physiology term from the Phenotype Ontology; and (d) Ageing-related genes: in this type of dataset each feature represents a gene in the GenAge [25] or the GenDR [26] database, and it takes the value 1 or 0 to indicate whether or not the compound associated with the current instance interacts with at least one protein encoded by a gene in GenAge or GenDR.

Note that the first feature type, protein interactors, represents a ‘direct’ property of a compound (drug), whilst the latter three feature types represent ‘indirect’ properties of a compound, in the sense that they are associated to the protein interactors and, by extension, to the compounds. To the best of our knowledge, of these four feature types, only GO terms have been used for predicting lifespan-extension compounds with machine learning algorithms [17, 18], and the other three feature types are novel contributions in this context.

As shown in Figure 1B, 1C, the GO term annotations and the physiology phenotype annotations datasets have features that are hierarchically related via a generalization-specialisation relationship. That is, if an instance (compound) takes the value 1 for a GO term or physiology phenotype feature, that instance will also take the value 1 for ancestors of that GO term or phenotype feature in the corresponding hierarchy, where the ancestors represent more general properties than their corresponding descendant features in the hierarchy.

For each feature type, we created two versions of a dataset. In “version 1”, every compound-protein interaction stored on the STITCH database is used to create predictive features, regardless of that interaction’s confidence score in the STITCH database. In “version 2” of a dataset, only compound-protein interactions with a confidence score of at least 45% in STITCH are used to create predictive features – this confidence-score threshold was also used in [17], and it represents a roughly ‘medium’ degree of confidence. See Section 4.1 for more details on the confidence-score threshold and dataset creation. Note that these two dataset versions represent different trade-offs between the quantity and the quality of the data available for training machine learning models, as follows. The version-1 datasets have more features, which provides more information for learning; but some of those features have a relatively low confidence score (the minimum confidence score for protein interactions in STITCH is 15%). The version-2 datasets have less features, providing less information for learning, but that information is more reliable. Hence, to investigate this trade-off, we have performed experiments with both versions of the datasets, for each type of predictive feature.

Table 1 shows the number of compounds (instances) and features (variables), as well as the class imbalance details, for each of the 8 datasets prepared for this study. The two dataset versions for each of the four feature types are denoted by the suffix “_1” or “_2” at the end of the datasets’ names. The class imbalance ratio is calculated by dividing the number of majority-class instances (compounds that are not associated with ageing, the negative class) by the number of minority class instances (ageing-related compounds, the positive class). The imbalance ratio represents how many instances of the majority class the dataset has for each instance of the minority class.

**Table 1:** Description of the datasets used in this study.

**Dataset name**	**Compounds**	**Features**	**Class imb. ratio**
Interactors_1	1120	5607	3.42 (22.6% pos.)
Interactors_2	1059	2563	3.46 (22.4% pos.)
GOTerms_1	1120	7588	3.42 (22.6% pos.)
GOTerms_2	1059	5572	3.46 (22.4% pos.)
Phenotypes_1	1103	1258	3.38 (22.8% pos.)
Phenotypes_2	910	1008	3.36 (22.9% pos.)
GenAge_1	1042	346	3.34 (23% pos.)
GenAge_2	719	139	3.46 (22.4% pos.)

Most of our datasets have a high number of features in comparison to the number of instances, which can hinder the performance of classification algorithms. Thus, we applied feature selection (FS) methods to each dataset in a data pre-processing phase, before using a classification algorithm to learn a predictive model from the data.

We performed experiments comparing six different types of FS methods. Five of these are based on well-established FS methods that follow the filter approach [27, 28], where a FS method measures the degree of association between each feature and the class variable and then selects the top-ranked features based on those measures – see Section 4.2 for details. The experiments also included ensemble versions of those filter methods, called filter ensembles, which combine the outputs of many runs of a filter method in a way that mitigates the problem of class imbalance in our datasets in order to improve robustness and predictive accuracy, as explained in Section 4.3. The sixth type of FS method is a novel FS approach proposed in this paper (Section 4.5), called Auto-Filter, that automatically performs a data-driven selection of the best filter or filter ensemble method for the input data, from a set of pre-defined candidate filter methods (Section 4.2) or filter ensemble methods (Section 4.3). The Supplementary Material for this paper has the detailed results of this comparison of FS methods.

The remainder of this Section is divided into three subsections. In Subsection 2.1 we discuss the predictive accuracy results for the experiments with all the created datasets. In Subsection 2.2 we interpret the biological meaning of the results for the best predictive (classification) models. In Subsection 2.3 we identify the most promising novel compounds for extending *C. elegans*’s lifespan, as predicted by the best models. In each of these subsections, we first discuss the results for the experiments with the version-1 datasets (using all compound-protein interactions in STITCH to create the predictive features), and then discuss the results for the experiments with the version-2 datasets (using only more reliable compound-protein interactions to create the features).

### Predictive accuracy results

All models discussed in this Section were created using the following experimental setup. The classification models were trained using a Random Forest (RF) algorithm, which is among the top-performing classification algorithms in general [29, 30] and is very popular in bioinformatics, and has also been used in previous studies for predicting lifespan-extension compounds [17–19]. The RF has the advantage of facilitating an indirect analysis of feature importance, which can be useful for detecting highly predictive features for a given classification problem, as shown later in Section 2.2. More specifically, we used the ‘Balanced Random Forest’ method [31] to cope with the class-imbalance issue in our datasets (see Section 4.6 for details).

As the RF algorithm has an embedded feature selection process, it is considered to be robust against datasets with a large number of features (like our datasets), so it is possible that performing FS in a data pre-processing step would not have a positive impact on the predictive accuracy of the resulting RF classifier. Therefore, we compared the results of a RF classifier trained using the candidate filter methods selected in our study, including the proposed Auto-Filter approach, against a Baseline RF classifier using all the original features, i.e. not performing any FS prior to training the classifier.

The predictive accuracy of the learned Random Forest classifiers was measured by the popular Area Under the ROC curve (AUC) measure, using a standard 10-fold cross-validation procedure [32]. The AUC measure takes values in the range [0..1], where 0.5 is the expected score for randomly guessing the class labels and 1 would be the score of a perfect classifier. We report the median AUC results over the 10 test folds of the 10-fold cross-validation, since the median is more robust to outliers than the mean.

The first set of experiments was conducted on the version-1 datasets, whose features are based on all compound-protein interactions stored in STITCH. Table 2 shows a comparison of the median AUC results for these experiments, between a baseline Random Forest (RF) model (i.e., a RF model where no feature selection was used prior to training the model) and the best RF model, obtained by training the model with the filter method that led to the highest AUC value on the training set. As mentioned earlier, the complete results from this comparison, including all filter results, is available at the Supplementary Materials file.

**Table 2:** Results for version-1 datasets: median AUC of the baseline RF model vs best RF model.

**Dataset**	**Baseline**	**Best model (best filter)**
Interactors_1	0.717	0.801 (Auto-Filter Ensemble)
GOTerms_1	0.767	0.818 (Auto-Filter Ensemble)
Phenotypes_1	0.741	0.761 (Ensemble of Decision Stump Filters)
GenAge_1	0.683	0.757 (Single Decision Stump Filter)

After evaluating the classification models using the cross-validation approach, we performed a statistical analysis to investigate whether the RF models trained with the features selected by the best filter method significantly outperforms the baseline RF model (with no feature selection in a pre-processing step), for each dataset. We applied a two-tailed Wilcoxon signed-rank test [33] (a non-parametric test that does not make assumptions about the data distribution) comparing the AUC results of each fold of the cross-validation process pairwise, meaning the same training and test folds were used for each pair of classifiers we compared. Note that this means that the sample size for the Wilcoxon signed-rank test is 10, corresponding to the results on the 10 test sets of the 10-fold cross-validation procedure. None of the 4 comparisons yielded significant results (p-values 0.275, 0.625, 0.921 and 0.275 for Interactors_1, GOTerms_1, Phenotypes_1 and GenAge_1 datasets respectively), meaning we could not reject the null hypothesis that the methods’ performances are statistically equivalent at the standard significance level of 5%.

However, considering that the difference between the median AUC results was relatively high in some cases in Table 2 (e.g., 8.4% for the Interactors_1 dataset), it is possible that the small sample size (10) used by the Wilcoxon signed-rank test had too much influence on the results of the statistical test, since the p-values computed by statistical significance tests are quite sensitive to the sample size, and lack of statistical significance does not mean lack of biological relevance [34–38]. Thus, we also calculated a measure of the ‘effect size’ for the differences of AUC values between each pair of methods in Table 2, which is a measure much less sensitive to sample size and is thus more suitable for identifying differences that are relevant in practice [34, 39], particularly when using a small sample size.

More precisely, we calculated the popular Cohen’s *d* measure of effect size [34] for each dataset, as an approach for investigating the difference between the AUC values of the Baseline RF and the RF trained with the features selected by the best filter method, for each dataset (feature type). The effect sizes are usually classified into small (*0.2* ≤ *d < 0.5*), medium (*0.5* ≤ *d < 0.8*) and large (*d* ≥ *0.8*), reflecting how apparent the difference between the groups is (with *d < 0.2* indicating an irrelevant or negligible effect).

For our comparisons of the results in Table 2, we found *d* = 0.593 for the Interactors_1 dataset (medium effect), *d* = 0.272 for the GOTerms_1 dataset (small effect), and *d* = 0.499 (borderline small, nearly medium effect) for the GenAge_1 dataset, which indicate a relevant difference in AUC values for these three datasets. Only the Phenotypes_1 dataset had *d* = 0.005, which indicates that the differences in the AUC values of the two approaches are negligible.

The second set of experiments was conducted on the version-2 datasets, created using only compound-protein interactions with a confidence score (in STITCH) above or equal to the threshold of 45%, representing a roughly ‘medium’ degree of confidence. Table 3 shows a comparison of the median AUC results for these experiments.

**Table 3:** Results for version-2 datasets: Median AUC of the baseline RF model vs best RF model.

**Dataset**	**Baseline**	**Best model (best filter)**
Interactors_2	0.747	0.752 (Information Gain, Ensemble Filter)
GOTerms_2	0.765	0.772 (Decision Stump, Ensemble Filter)
Phenotypes_2	0.716	0.718 (Information Gain, Ensemble Filter)
GenAge_2	0.701	0.72 (Asymmetric Optimal Prediction, Ensemble Filter)

In this set of experiments the combined use of RF and filter methods also outperformed the baseline RF algorithm in all cases, but the difference in performance was smaller. As with the previous set of experiments, we used the two-tailed Wilcoxon signed-rank test, followed by Cohen’s *d* measure of effect size, to compare the performances of the best RF model (with the best filter) to the baseline RF model, for each dataset. None of the Wilcoxon test results were significant (p-values 0.921, 0.921, 0.322 and 0.284 for the Interactors_2, GOTerms_2, Phenotypes_2 and GenAge_2 datasets, respectively), so the null hypothesis that the AUC results between the two models is equivalent cannot be rejected at the standard significance level of 5%. Regarding the effect size analysis, the only dataset where a small effect was detected was GenAge_2 (*d* = 0.451). The others did not have significant effects (*d* = 0.018, *d* = 0.019 and *d* = 0.0071 for Interactors_2, GOTerms_2 and Phenotypes_2 datasets, respectively).

These statistical results are not unexpected, considering that the median AUC values of the best RF models (with the best filters) are closer to the baseline RF model in this set of experiments with the version-2 datasets, by comparison with the results for the version-1 datasets. Note that the confidence-score threshold applied during the creation of the version-2 datasets filters out less reliable features, which simultaneously strengthens the use of the baseline RF algorithm and reduces the positive impact of applying filters to the dataset in a preprocessing step. Nevertheless, over all four datasets in Table 3, the ensemble filter methods consistently achieved a marginally better predictive accuracy.

Next, we discuss the effect of the type of feature on the predictive accuracy of the classifiers for each dataset version.

Across all RF classifiers for the version-1 datasets, the best and second best classifiers (with the highest median AUC values) were the RF classifies trained with the features selected by the Auto-Filter approach for the GOTerms_1 and the Interactors_1 datasets, respectively. In the experiments with the version-2 datasets, we again see that the best and second best models were produced with the GOTerms_2 and Interactors_2 datasets, respectively.

In addition, for both versions of the datasets, the feature type leading to the smallest AUC value was GenAge. This seems at first glance surprising, since this is the only feature type that directly represents background knowledge on ageing. However, the lower performance of this feature type can be explained by the fact that its number of features is much smaller than the number of features for the other feature types (i.e. it provides much less information for learning), as shown in Table 1.

Although at first glance the version-1 datasets led to higher AUC values than the version-2 datasets (comparing the results in Tables 2, 3), the comparison of the AUC values for these two dataset versions is complicated by the fact that they represent different trade-offs between the quantity and the quality of the data (predictive features), as mentioned earlier. Even though we used cross-validation to compute AUC values, and cross-validation is a very well-established methodology for measuring generalisation performance, the cross-validation procedure still has a limitation, as follows. When there is some noise in the entire dataset, the same kind of noise will in general be present in both the training and the testing sets. Hence, during cross-validation, the model learned from the training set can incorporate some relatively spurious patterns capturing some noise in the training set, and those patterns could broadly hold on the test set, so that learning those patterns could artificially somewhat increase the apparent predictive accuracy (AUC value) on the test set. In the case of our experiments, the version-1 datasets in principle have indeed somewhat more noise than the version-2 datasets, since some feature values in the former are less reliable.

Hence, as a stricter evaluation of the predictive performance (generalisation ability) of the best models learned from the version-1 and version-2 datasets, we also evaluate those best models in an “external” validation dataset, which is completely independent from the dataset used for cross-validation. Since we used all compounds with data for *C. elegans* in DrugAge for performing the cross-validation experiments, we have to use, as an external validation dataset, compounds with data for another species in DrugAge. We chose *D. melanogaster* as the model organism for this external validation dataset because it is the second most common model organism on DrugAge, after *C. elegans*. Naturally, this is not ideal because the effects of a compound on longevity can vary significantly between organisms. Thus, any conclusion from this additional experiment is predicated on the major assumption that a compound’s effect on *D. melanogaster’s* lifespan would be broadly similar to its effect on *C. elegans*’ lifespan.

For evaluation on the external *D. melanogaster* dataset, we selected four models in total, the two best models for each version of the *C. elegans* datasets – i.e., the models trained with protein interactors and GO terms, with their best filter method, for each dataset version. Hence, we used those four models, trained using *C. elegans* data, to predict the class label of 300 instances in the corresponding *D. melanogaster* datasets, as external validation datasets. Each of the four *D. melanogaster* datasets was created using the same features used by the corresponding best model for *C. elegans* dataset and using the class labels defined by the data for *D. melanogaster* on DrugAge.

For the version-1 datasets, with more features but less reliable features overall, the Interactors_1 model learned from *C. elegans* data had 0.551 median AUC on the *D. melanogaster* dataset (down from 0.801 on the *C. elegans* dataset, and the GOTerms_1 model had 0.523 (down from 0.818). For the version-2 datasets, with less features but more reliable features overall, the Interactors_2 model learned from *C. elegans* data had 0.642 median AUC on *D. melanogaster* data (down from 0.752 on *C. elegans* data), and the GOTerms_2 model had 0.672 (down from 0.772).

In summary, when using the best models trained on *C. elegans* data to classify *D. melanogaster* data (as a more challenging evaluation of generalisation ability), the reduction of accuracy we observed in the two best models learned from version-1 datasets was between 25% and 30%, while the reduction for the two best models learned from version-2 datasets was about 10%. Although a substantially reduced predictive performance was of course expected in these tests, considering the issue of using a different model organism in the external validation dataset, the much larger performance drop for the two best models learned from version-1 datasets (with more data but overall less reliable data) can be considered an indicator of overfitting of those models to the datasets they were trained on. Hence, the two best models learned from version-2 datasets (with less data but overall more reliable data) had better generalisation ability on the external validation datasets. These results also show the importance of using an external validation dataset, as a more challenging measure of generalisation ability, by comparison with cross-validation.

### Analysis of feature importance in the best predictive models

Supervised machine learning models, in addition to being tools for predicting target variables, reflect patterns in the data used to train them. Interpreting a classification model, by identifying how its predictions are made, can help the user both check the internal consistency and biological validity of the decisions made by the algorithm and find interesting patterns that may spark new research directions.

Random Forest models are ensembles of decision trees. Although a single decision tree can be directly interpreted (if it is not too large), directly interpreting each random tree in the forest is not feasible, due to the large number of trees. As an alternative, we can calculate a feature importance measure, which allows the user to see which features are considered most important for classification across all trees in the forest.

In this Subsection we analyse the top features of our most successful predictive model for each of the two dataset versions, where both models were trained with the GO Terms feature set: the model for the GOTerms_1 dataset and the model for the GOTerm_2 dataset.

For this feature importance analysis, we trained new models using the entire datasets (no training and test set division), to ensure the analysis would consider all data available. The Balanced Random Forest method was used again to deal with class imbalance, in order to avoid a bias in favour of the majority class in the predictions.

The metric of feature importance used in this analysis was the Gini Importance Measure (GIM). The GIM is the average reduction in Gini Index over all nodes in the decision tree which use that feature for branching, over all decision trees in the forest [40, 41]. The main drawback of this metric in general is a bias in favour of features with many possible values. However, this drawback does not occur in our case as all predictive features are binary. The GO terms selected in this analysis are essentially the features that are most relevant for discriminating between positive-class (lifespan-extending) and negative-class (non-lifespan-extending) compounds, for these models.

Table 4 shows the top 10 features based on GIM for the GOTerm_1 model. In this Table, the last column shows the proportion of positive-class instances (i.e., lifespan-extending compounds) among the instances which take the value ‘1’ for the feature – i.e., among all compounds annotated with the corresponding GO term. For example, for the first row in Table 4, there are 17 compounds annotated with the GO term ‘Respiratory chain complex II assembly’, out of which 15 (88.2%) are positive-class compounds. Note that there are two complementary ways for a feature to be one of the most relevant features: the feature can be a strong predictor of the positive class (i.e., the positive-class proportion is very high) or a strong predictor of the negative class (i.e., the positive-class proportion is very low). Only two features in Table 4 are associated with the negative-class – i.e., compounds taking the value ‘1’ for such features are in general negative-class compounds (their positive-class proportion is 0). Note that the features in Table 4 include only the Molecular Function and Biological Process categories of GO terms, excluding Cellular Component GO terms, because we found that the selected top features in this latter category were redundant with respect to one of the top Molecular Function terms.

**Table 4:** The most important features in the RF classifiers trained with the GOTerms_1 dataset and feature selection performed using the Auto-Filter approach.

**GO term**	**Type**	**Name**	**Positive class proportion**
GO:0034552	Biological process	Respiratory chain complex II assembly	88.2% (15/17)
GO:0034553	Biological process	Mitochondrial respiratory chain complex II assembly	88.2% (15/17)
GO:0015689	Biological process	Molybdate ion transport	80% (8/10)
GO:0015098	Molecular function	Molybdate ion transmembrane transporter activity	80% (8/10)
GO:0004965	Molecular function	G protein-coupled GABA receptor activity	0% (0/29)
GO:0007214	Biological process	Gamma-aminobutyric acid signalling pathway	0% (0/29)
GO:0015288	Molecular function	Porin activity	81% (13/16)
GO:0005052	Molecular function	Peroxisome matrix targeting signal-1 binding	80% (12/15)
GO:0016560	Biological process	Protein import into peroxisome matrix, docking	80% (12/15)
GO:0015098	Molecular function	Molybdate ion transmembrane transporter activity	80% (8/10)

The GO term features (in Table 4) associated with longevity drugs reflect processes commonly associated with aging and those more often targeted in longevity pharmacology. The mitochondrial respiratory chain complex is the top GO term, reflecting that this complex has been associated with aging and is often targeted in longevity pharmacology [15]. Other GO terms, like porin activity, are also likely related to mitochondria. Therefore, as expected, our results largely include processes previously associated with aging. In addition, we found terms (e.g., “G protein-coupled GABA receptor activity” and “Gamma-aminobutyric acid signalling pathway”) associated with the negative class; that is, terms predictive that a drug will not extend lifespan.

For the model trained with the version-2 dataset, we excluded both molecular function and cellular component GO Terms of the top features analysis, as they were too broad to add valuable insight in this analysis. Thus, Table 5 shows the top 10 biological process GO Terms with the highest GIM, from the best Random Forest model trained with the GOTerms_2 dataset.

**Table 5:** The most important biological process GO terms in the RF classifiers trained with the GOTerms_2 dataset and feature selection performed using the decision stump ensemble approach.

**GO term**	**Name**	**Positive class proportion**
GO:0051246	Regulation of protein metabolic process	40.8% (127/311)
GO:0071868	Cellular response to monoamine stimulus	11.7% (46/393)
GO:1903350	Response to dopamine	40.8% (127/311)
GO:0006749	Glutathione metabolic process	59.3% (70/118)
GO:0009636	Response to toxic substance	45.5% (106/233)
GO:0071870	Cellular response to catecholamine stimulus	11.7% (46/393)
GO:0006950	Response to stress	25.3% (191/755)
GO:0006091	Generation of precursor metabolites and energy	53.0% (80/151)
GO:0000003	Reproduction	26.3% (174/662)
GO:0046395	Carboxylic acid catabolic process	48.4% (92/190)

The main GO term associated with the positive class (lifespan extension) is ‘Glutathione metabolic process’ – almost 60% of the compounds interacting with a protein annotated with this GO term belong to the positive class. The glutathione metabolic process is a complex and tightly regulated system that plays an important role in maintaining cellular redox homeostasis, detoxification, and immune function [42]. Note that most of the other GO terms in Table 5 have a positive-class proportion smaller than 50%. However, recall that the ‘baseline’ positive-class proportion, considering all compounds in the full dataset, is just about 22% (Table 1, last column). In this context, GO terms with a positive-class proportion in Table 5 in the range 40%-50% can also be considered to be associated with the positive class, in the sense that proteins interactors annotated with such GO terms lead to a major increase the corresponding probability that a compound is predicted to belong to the positive class. Hence, the GO term ‘Response to toxic substance’ (with a positive-class proportion of about 45%) also fits our current knowledge of longevity assurance mechanisms involving detoxification processes, which interestingly include anti-oxidant enzymes like glutathione [43]. These processes fit current knowledge of pathways related to aging in model organisms related to oxidative stress, in particular in invertebrate models [44]. They also reflect a long-term trend in the anti-aging field, and more recently in longevity biotech, employing protective antioxidant compounds [12].

### Identifying the most promising novel compounds for lifespan extension

In this Subsection we identify the compounds, from a list of ~1300 unlabelled compounds from DrugBank (ignoring compounds that are isomers of compounds on DrugAge), with the highest probability of being classified as members of the life-extension class (positive class) by the best classifier from each of the two dataset versions, where both best models were trained with GO term features. We then calculated how many of these compounds have a large majority of positive-class “neighbours” in our DrugAge dataset, using this as a criterion for selecting the most promising novel compounds. Therefore, the compounds selected in this analysis represent potentially novel compounds for extending *C. elegans*’ lifespan, although whether or not they really have this effect needs to be validated by proper biological experiments, of course, which is left for future research.

The datasets of unlabelled compounds were created using the DrugBank database (version 5.0, downloaded in June 2022) [45], an online database of drugs and drug targets. The 10 top most promising compounds for the GOTerms_1 model are listed in Table 6.

**Table 6:** The 10 compounds with highest positive-class prediction probability, from the GOTerms_1 dataset classifier.

**DrugBank code**	**Compound**	**Predicted probability of positive class**
DB00157	**NADH**	96.8%
DB01992	Coenzyme A	94.8%
DB00131	Adenosine phosphate	92.8%
DB15998	**Potassium hydrogen DL-aspartate**	92.2%
DB00171	ATP	92%
DB00115	Cyanocobalamin	91.6%
DB04137	Guanosine-5’-Triphosphate	91.6%
DB13949	**Ferric cation**	91.4%
DB01082	**Streptomycin**	91.2%
DB14577	Calcium cation	90.4%

As a secondary criterion for selecting promising novel compounds, we measured the similarity of each of the top DrugBank compounds to the compounds in our original dataset. This allowed us to determine how often their ‘neighbours’ (i.e., the most similar compounds in our dataset regarding their feature values in the dataset) are labelled as lifespan-extension compounds (positive-class). We used the Jaccard coefficient [46] to calculate the similarity between compounds – a measure of similarity between binary sets that only considers positive matches (ignoring matches of ‘0’ values), widely used in biology studies.

This criterion was chosen because, intuitively, the DrugBank compounds with many positive-class neighbours in our dataset, in addition to having a high probability of positive-class prediction by our best classification models, are the best choices for possible novel compounds for longevity research. Thus, we set a cut-out point of at least 80% positive-class neighbours, from the 20 most similar compounds in our original datasets, as our second selection criterion in this analysis. Based on this we selected four compounds to focus on, out of all compounds in Table 6: NADH (16/20 positive-class neighbours), Potassium hydrogen DL-aspartate (17/20), Ferric cation (17/20) and Streptomycin (17/20). The other compounds in Table 6 did not reach our threshold of 80% positive-class neighbours and, although still relevant as possible novel compounds, will not be discussed in detail.

The top predicted new longevity compound in Table 6 is NADH, the reduced form of NAD+, which is involved in metabolism and redox reactions. There has been significant interest into NAD+ and aging, including into NAD+ enhancers as a potential therapy [15, 47].

Interestingly, Potassium hydrogen DL-aspartate has not, to our knowledge, been studied in the context of longevity; but has been shown in cells to inhibit damage and apoptosis from oxidative stress [48], and thus may be interesting to study in the context of longevity. Sun et al. [48] suggests that L-aspartic acid potassium salt protects from apoptosis and damage. This compound is chiral, meaning that there are two mirror images (isomers) of that same compound available. This means that in an environment where there are chiral targets (e.g., proteins) or other chiral molecules, this can affect properties that the molecule may have. Our best classifier from the version-1 datasets suggests that Potassium hydrogen DR-aspartate (a mixture of both isomers) may have a role to play in longevity, supported by this data on L-aspartic acid potassium salt (a single isomer of the same compound).

Ferric cation also, to our knowledge, has not been studied in the context of aging, although it is interesting to note that iron metabolism has been associated with aging [49].

Also noteworthy, Streptomycin is an antibiotic often used in *C. elegans* culture. If Streptomycin were to extend lifespan in worms then it could be a potential source of bias in longevity studies, hence further studies are warranted.

Importantly, the results in Table 6 refer to the models created with our GOTerms_1 dataset, which by design includes some data from low-confidence compound-protein interactions (i.e., interactions for which there is relatively little evidence on the STITCH database). Therefore, this should be taken into account when interpreting these results.

Table 7 shows the top-12 compounds for the same analysis, done using the best classifier for the version-2 datasets, i.e. the classifier trained with the GOTerms_2 dataset. Recall that this classifier was trained with more reliable data and obtained much better generalisation performance on an external validation dataset than the best classifier for the version-1 datasets. The dataset of unlabelled compounds has the same instances from DrugBank described earlier.

**Table 7:** The 12 compounds with highest positive-class prediction probability, from the GOTerms_2 dataset classifier.

**DrugBank code**	**Compound**	**Predicted probability of positive class**
DB01992	Coenzyme A	98.40%
DB04854	Febuxostat	97.93%
DB01685	Topiroxostat	97.93%
**DB00157**	**NADH**	**95.00%**
DB15412	LB-100	93.13%
DB08822	Azilsartan medoxomil	93.13%
DB11191	Cobamamide	89.60%
DB17293	Hiltonol	89.20%
DB00905	Bimatoprost	88.44%
DB07187	CP-744809	88.44%
**DB00325**	**Nitroprusside**	**88.40%**
**DB03147**	**Flavin adenine dinucleotide**	**87.40%**

Based on the Nearest Neighbour analysis to determine the most promising novel compounds for lifespan extension (at least 80% positive-class nearest neighbours out of the 20 most similar compounds in the original dataset), we selected three compounds from Table 7: NADH (16/20), which also appears in Table 6, Nitroprusside (16/20) and Flavin adenine dinucleotide (16/20).

Nitroprusside is an antihypertensive medication which, as recorded in the DrugBank database, is an agonist of NPR1 (atrial natriuretic peptide receptor 1), which is a receptor for peptides which are vasoactive hormones playing a key role in cardiovascular homeostasis. NPR1’s Biological Process GO term annotations in UniProt include, among others: “regulation of blood pressure”, “regulation of vascular permeability”, “negative regulation of angiogenesis” and “negative regulation of cell growth”. To our knowledge, nitroprusside has not been studied in the context of aging or longevity. One recent study, however, found two antihypertensive medications to extend longevity in C. elegans [50]. Furthermore, in recent *in vivo* experiments with Npr1 knockout mice, Npr1^+/–^ mice has exhibited vascular aging [51]. As recorded in DrugBank, Flavin adenine dinucleotide (FAD) is a coenzyme form of vitamin B2 used in clinical conditions associated with vitamin B2 deficiency. FAD is a redox-active coenzyme that has also not, to our knowledge, been studied in the context of aging, although it extended lifespan in an frataxin deficiency model in C. elegans [52]. In addition, Vitamin B2 is essential for *C. elegans* growth [53]. FAD has 86 targets recorded in DrugBank, indicating the complexity of its effects on metabolism. Disorders of FAD metabolism are reviewed in [54]. In experiments with male Wistar rats and spontaneously hypertensive rats (SHRs) treated with FAD for 8 weeks, FAD ameliorated vascular remodelling in SHRs, and was suggested as a new potential treatment for hypertension and vascular remodelling [55].

## CONCLUSIONS

We created datasets for predicting whether or not a compound extends the lifespan of *C. elegans*, using data from the DrugAge database to assign a positive or negative class label to each compound, depending on whether or not the compound is recorded in DrugAge as significantly extending *C. elegans*’ lifespan by at least 5%. The datasets use four different types of predictive features, based on compound-protein interactions, interactions between compounds and proteins encoded specifically by ageing-related genes, and two types of terms annotated for proteins targeted by the compounds, namely Gene Ontology (GO) terms and physiology terms from the WormBase’s Phenotype Ontology. For each of these four feature types, we created two versions of a dataset. The version-1 datasets were created using all compound-protein interactions from the STITCH database to create features, a very inclusive approach. The version-2 datasets were created using only the compound-protein interactions in STITCH which have at least a roughly ‘medium’ degree of confidence, a stricter approach. Hence, version-1 datasets have more features but less reliable features, whilst version-2 datasets have less features but more reliable features; i.e., these datasets represent different trade-offs in the quantity and quality of data available for machine learning algorithms.

To analyse these datasets we used a combination of feature selection methods in a data pre-processing phase and the well-established random forest algorithm for learning a predictive model from the selected features. In terms of predictive power of the different types of features, in the experiments with both versions of datasets, the best model (regarding predictive performance) was learned using GO terms as predictive features. We also evaluated those two best models using GO terms as features (one for each dataset version) on an external validation dataset with *D. melanogaster* data. This experiment was a more challenging evaluation of the generalisation ability of the predictive models, since they were learned from *C. elegans* data and used to classify data from a different species. Hence, predictive performance was of course expected to decrease, but the observed decrease was much smaller for the best model learned from the version-2 dataset than for the best model learned from the version-1 dataset. Hence, the version-2 dataset of GO terms as features showed better generalisation ability on external validation data; i.e., in this experiment having higher quality data was more beneficial than having a larger quantity of data.

In addition, we used a feature importance measure to identity the most relevant features in the best random forest model for each dataset version. Among those top-ranked features, there are several GO terms that are known to be associated with the ageing process, particularly involving the mitochondrial respiratory chain complex and longevity assurance mechanisms like detoxification and glutathione, which are often targets for longevity drugs.

Furthermore, we identified the most promising novel compounds for extending *C. elegans* based on the predictions of the best learned random forest models – i.e., compounds from the DrugBank database (not included in the data used to train the classifiers) that were predicted with a very high probability to be positive class (extending lifespan) compounds.

In the experiments with version-1 datasets, the most promising novel compounds (which have not been investigated in the context of ageing yet, to the best of our knowledge) included Potassium hydrogen DR-aspartate and streptomycin. Potassium hydrogen DR-aspartate is a mixture of two isomers, and the hypothesis of its potential pro-longevity effect in *C. elegans* is supported by the data for a single isomer of that compound (L-aspartic acid potassium salt). Streptomycin is an antibiotic often used in *C. elegans* culture, and so, if further research confirms that this compound really extends the lifespan of *C. elegans in vivo*, this would show an important source of currently undetected bias in longevity experiments with *C. elegans*.

In the experiments with version-2 datasets, some of the most promising novel compounds include nitroprusside, flavin adenine nucleotide and NADH. Nitroprusside, which is a powerful vasodilator used to treat hypertension, is an agonist for *Npr1*, a gene implicated in vascular aging in mice. Flavin adenine dinucleotide is a coenzyme form of vitamin B2, which is one of the vitamins essential for *C. elegans* growth. In addition, NADH, the reduced form of NAD+, is involved in metabolism and redox reactions, and it was among the most promising novel compounds for extending *C. elegans* lifespan in the experiments with both the version-1 and version-2 datasets.

Future research will involve lab experiments with *C. elegans* in order to try to confirm these computational predictions.

## MATERIALS AND METHODS

### Dataset preparation

We created four types of datasets, all consisting of instances representing chemical compounds (or drugs), and all with the same definition of positive and negative class labels, but different types of predictive features (variables). In all datasets, the positive-class instances consist of drugs or compounds whose administration led to a statistically significant average increase of at least 5% of *C. elegans*’ lifespan, as recorded in the DrugAge database (Build 4) [13]. The DrugAge database collects information of potentially life-extending compounds, based on publications reporting wet-lab experimental results (Website: genomics.senescence.info/drugs).

The list of negative-class instances (i.e., compounds found to have no significant positive impact on *C. elegans*’ lifespan) was taken mainly from the Supplementary Material provided in a previous study [17] that created a similar dataset, with two extensions, as follows. First, some compounds were included in DrugAge for having lifespan-increasing effects on other organisms, but their impact on *C. elegans*’ lifespan was negative, so these compounds were used as negative-class instances. Second, some of the negative-class instances from the list in [17] were updated as positive-class based on more recent information in DrugAge, as the previous list was based on information from 6 years ago.

As mentioned earlier, we used four types of predictive features (each generating two datasets), namely features based on Gene Ontology (GO) terms, drug (compound)-protein interactions, interactions between compounds and proteins encoded specifically by ageing-related genes, and physiology terms from a Phenotype Ontology for *C. elegans*. Thus, all predictive features in our datasets are related to the proteins that interact with each compound, namely: the protein interactors themselves, the GO term annotations and the Physiology Phenotypes associated with those interacting proteins, and whether the protein interactors of a compound are coded by an ageing-related gene. Notably, two of the three most related works [17–19] (applying machine learning to DrugAge data) also use GO term features, namely [17, 18], but none of those three works used protein interactors, phenotypes or ageing-related genes as predictive features.

Our source of protein-compound interaction was the STITCH database (version 5.0, downloaded in 11-2021. Website: http://stitch.embl.de/) [56], a database of interactions between chemicals and proteins. We discarded all compounds that either were not found on STITCH or did not have any information of protein interactions stored there. In particular, we removed from our initial list of compounds (instances) all the entries for plant extracts that are not used commercially as drugs, since there is no entry in STITCH for such extracts. This filtering process caused our sample size to be reduced by about 25% compared to the original dataset in [17], arguably making the prediction problem more difficult. However, this was necessary for the types of predictive feature used in our datasets, as they are all based on protein interactors, and to compensate for the dataset reduction we got the benefit of creating datasets where all features are biologically interpretable, as mentioned earlier. After this instance (compound) filtering process, we obtained an initial dataset with 1120 instances, 253 (22.6%) of which refer to lifespan-extending (positive-class) compounds in *C. elegans*, with the remaining 867 (77.4%) being negative-class compounds. With this initial set of instances, we created in total eight datasets, as follows.

We created two dataset versions for each of the four feature types, based on the degree of confidence of the compound-protein interactions used as a basis for creating the predictive features in our datasets. The STITCH database sets a score value for each of its chemical-protein interactions, which is based on the amount of evidence available for that interaction. According to the database documentation, this score can be interpreted as a confidence indicator for how likely an interaction is to be true. In our case, we created a first set of datasets (version-1 datasets) where we did not set a minimum confidence-score threshold, so that every compound-protein interaction in STITCH was used to create predictive features for our datasets. Note that, as STITCH stores interactions with at least 15% confidence score, that is the effective minimum confidence-score threshold for the version-1 datasets. Then, we created a second set of datasets (version-2 datasets) using the same confidence-score threshold used in [17], 45% minimum confidence. The version-2 datasets have fewer features (less data for learning), but with the trade-off that their data is more reliable, by comparison with the more inclusive version-1 datasets.

After the initial creation of each dataset, we applied a simple frequency-threshold filter to remove features with fewer than 10 instances with a ‘1’ value in the training set (all features in our datasets are binary, with ‘1’ indicating the presence of the feature). This frequency-threshold filter was applied to reduce the risk of overfitting, and it was applied at the start of the data pre-processing phase, i.e. before applying the feature selection filters. In addition, if the application of this frequency-threshold filter results in an instance having all its features taking the value ‘0’ (rather than ‘1’), that instance is also removed from the dataset. As a result, the final number of instances in each dataset (before running feature selection methods and the random forest algorithm) is given in Table 1.

### Protein interactors dataset

For this dataset, we created binary features that indicate whether or not a protein interacts with the current instance (compound). A similar dataset was used in [57]. The created version-1 dataset initially had 9880 unique protein interactors obtained from the STITCH database (version 5.0), which were reduced to 5607 predictive features, after applying the aforementioned simple frequency-threshold filter to avoid overfitting. For the version-2 of this dataset, the number of protein interactors was reduced from 7374 to 2563. The number of interactors associated with a given compound varies greatly, reaching over 1000 interactors for some well-known compounds.

### Gene ontology dataset

Expanding on the information from the previous dataset, we used the Gene Ontology (GO) terms [23, 58] associated with each of the protein interactors as binary features in a second type of dataset. There are three types of GO terms, reflecting different types of information about a protein’s functions, namely: biological process, molecular function and cellular component. All 3 GO term categories were used as predictive features in the dataset, for the sake of completeness.

Each binary feature indicates whether or not an instance (compound) is indirectly associated with a given GO term. More precisely, the feature value ‘1’ means that at least one of the proteins that interact with the compound (as recorded in the STITCH database) is annotated with the corresponding GO term. Conversely, the feature value ‘0’ means that none of the proteins interacting with the compound have been annotated with that GO term. The proteins’ GO term annotations were obtained using the *goatools* (version 1.1.6) Python library, from the Gene Ontology version 1.4, downloaded in 11-2021 (Website: geneontology.org). The created version-1 dataset initially had 9000 unique GO terms, which were reduced to 7588 predictive features after applying the simple frequency-threshold filter. The created version-2 dataset had 8364 unique GO Terms, which were reduced to 5572 predictive features after the same procedure.

### Phenotypes dataset

In this dataset, each binary feature indicates whether or not a compound (instance) is indirectly associated with a given Phenotype Ontology (Physiology) term in the WormBase database (release WS283, downloaded in 11-2021) [24] (Website: https://wormbase.org/tools/ontology_browser). More precisely, the feature value ‘1’ means that at least one of the proteins that interacts with the compound (as recorded in STITCH) is annotated with the corresponding Phenotype Ontology term; otherwise, the feature takes the value ‘0’. Note that the Phenotype Ontology has physiology and anatomical phenotypes, and we only used the physiology terms, because anatomical characteristics are not as relevant for our prediction problem (whether or not a compound’s administration extends *C. elegan*’s lifespan). The created version-1 dataset initially had 1783 physiology phenotype features, which were reduced to 1258 after applying the frequency-threshold filter. The created version-2 dataset had 1008 predictive features after applying the frequency-threshold filter.

### Age-related genes dataset

In this dataset, each predictive feature indicates whether or not a compound (instance) interacts with a given age-related gene. To create these features, we used the lists of *C. Elegans* genes in the GenAge [29, 59] (Build 20, downloaded in 11-2021; website: https://genomics.senescence.info/genes) and the GenDR [26] (Build 4, downloaded in 11-2021; website: https://genomics.senescence.info/diet) databases.

The GenAge database is a collection of genes from different organisms known to be associated with longevity and/or ageing. The GenDR database is a collection of genes specifically associated with dietary restriction (including caloric restriction), included in the definition of this feature type because this intervention is commonly associated with lifespan increase in multiple organisms, and some of those genes were not listed in GenAge.

Based on the proteins coded by these genes, we defined the value of their features using the list of protein interactors associated with each compound. If at least one of the interactors of a compound is coded by a given gene (feature), its feature value in the dataset is ‘1’, otherwise the value is ‘0’. The created version-1 dataset initially had 553 binary features, which were reduced to 346 features after applying the frequency-threshold filter. The created version-2 dataset initially had 353 features, which were reduced to 139 features after applying the frequency-threshold filter.

### Filter feature selection methods

Filter methods are a type of feature selection (FS) method used in a pre-processing phase of machine learning – before training the classification algorithm [60]. They calculate a score for each feature in the dataset, usually based on the distribution of its values in relation to the class label. Then, the top *k* (a user-specified parameter) features with the highest scores are kept, and all others are discarded. Note that filters are independent from the classification algorithm, in contrast to the more computationally expensive wrapper FS methods [60].

Filter methods differ mainly in terms of how they calculate the features’ scores, as there are various ways to measure feature importance, and no method is the best for all datasets. For our experiments in this study, we selected 5 different filter methods and, in addition to these, we developed a sixth (meta)-method called Auto-Filter, which automatically selects the best candidate filter method for each dataset using a data-driven approach. These filter methods are described next.

### Information gain

This filter calculates the score of a feature as the value of the Information Gain (reduction of Entropy) obtained by partitioning the instances of a dataset into subsets, based on the values of that feature. Notably, this measure is known to be biased in favour of features with many values [61], but this is not an issue in our case as all predictive features in our datasets are binary. The Information Gain is calculated for each feature *F* as follows.


$$Ent(C)=-\sum_{i=0}^{1}p(C_{i})\log_{2}p(C_{i})$$

$$Ent(C|F=j)=-\sum_{i=0}^{1}p(C_{i}|F=j)\log_{2}p(C_{i}|F=j)$$

$$\begin{array}{l} Ent(C|F)=Ent(C|F=0)\times W(F=0)+Ent(C|F=1)\\ \times W(F=1) \end{array}$$

IG(F)=Ent(C)−Ent(C|F)

Where *Ent(C)* is the entropy of the class labels on the training data, *p*(*C_i_*) is the empirical probability (relative frequency) of class *i* in the training set (as our classification problem is binary, the class is either *0* or *1*), and *Ent(C|F)* is the entropy of the class labels conditioned on the values of feature *F* on the training data. We calculate the entropy of the class labels before and after splitting the dataset using the current feature, i.e. calculating *Ent(C)* and *Ent(C|F)* respectively, the latter being a weighted sum of the Entropies of the class labels in both splits (data subsets where *F = 0* and *F = 1*), where the weights are the proportions of instances with *F = 0* and *F = 1* in the training data. The information gain is the difference between the entropy *Ent(C)* and the conditional entropy *Ent(C|F)*, with larger values indicating a greater reduction of class-label entropy, i.e. a stronger predictive power associated with the feature.

### The Chi^2^ (Chi-squared) statistic

The Chi^2^ test is a statistical hypothesis test used to get an estimation of the degree of association between two categorical variables (in our case, each predictive feature and the class variable). It compares the observed and expected frequencies of each combination of values of those two variables, and larger differences between these values indicate that the variables have a stronger association [62]. We used the value of the Chi^2^ statistic calculated by this test as the score for a filter method, as the higher this value gets, the greater the association between the feature and the class variable. The Chi^2^ score is calculated as follows.


$$\chi^{2}=\sum_{i}\sum_{j}\frac{{\left(O_{ij}-E_{ij}\right)}^{2}}{E_{ij}}$$

Where *O_ij_* represents the observed frequency of the co-occurrence of the *i*-th value of a feature and the *j*-th class label, i.e. the number of instances with the *i*-th feature value and the *j*-th class label in the training set, and *E_ij_* represents the expected value of that frequency of co-occurrence under the assumption that the feature and the class variable are statistically independent.

### Decision stump

A Decision Stump is the simplest version of a decision tree classifier, where the class label is decided based on a single node partition of the data, using a single feature. It usually does not have much predictive power on its own, but it can be used in other contexts such as providing a score for a filter method [63]. The score of a feature is calculated by training a decision stump classifier with a very narrow subset of the training data containing only that feature and the class variable, then evaluating the trained classifier on a subset of data which was not used for training (to estimate generalisation performance). In our experiments, the performance of a decision stump classifier was estimated by an internal 5-fold cross-validation procedure, applied to the training set only (i.e. not using the test set). Hence, the training set is divided into 5 folds and the decision stump classifier was trained 5 times, each time using a different fold as the ‘validation set’ (to estimate generalisation performance) and the other four folds as a ‘learning set’. We used the median of the five AUC values obtained by the decision stump classifier over the 5 validation sets as the score for this filter.

### Log odds ratio

**Figure 2:**
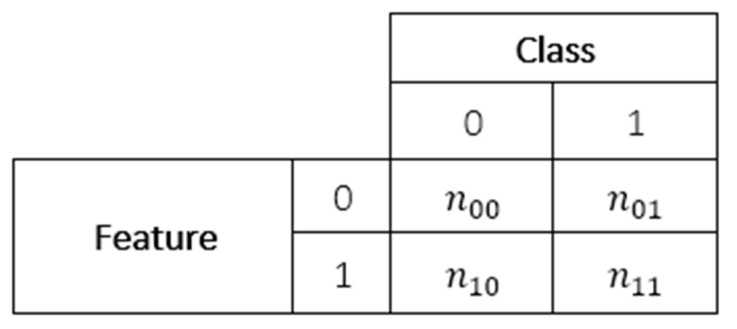
Structure of a contingency table.

The associations between two categorical variables, such as a binary predictive feature and the binary class variable in our datasets, may be displayed through a contingency table. In these tables each cell contains a count (*n*) or probability (*p*) of each combination of the values of the two variables, as shown in Figure 2. Some measures of association can be calculated based on this representation, including the Log Odds Ratio and the Asymmetric Optimal Prediction filters, described in this Subsection and the next.

The Odds Ratio [64] is a measure of association applicable to binary variables, which estimates the odds of an outcome based on the exposure (i.e., how much the odds of getting a ‘1’ value for a variable change based on the value of the associated variable). Odds values higher than 1 indicate an increased probability of success, and values lower than 1 indicate the opposite.

Smaller data samples cause the distribution of the Odds Ratio to be highly skewed. Thus, the natural logarithm of this measure, Log Odds Ratio, is used instead. The Log Odds Ratio between each feature and the class variable was used as a score for this filter method, calculated as follows.


$$\text{Log}\;\text{Odds}\;\text{Ratio}=\log\left(\frac{n_{00}\times n_{11}}{n_{01}\times n_{10}}\right)$$

### Asymmetric optimal prediction

The Asymmetric Optimal Prediction (AOP) is another measure of association between two categorical variables, with the distinction of measuring an asymmetric predictive relationship, i.e. measuring to what extent the value of variable *A* can be well predicted by the value of another variable *B*, regardless of strength of the converse type of prediction (predicting *B* from *A*) [65]. This is relevant because in the classification task we use the feature value to predict the class label, not vice-versa, therefore it can be beneficial to focus on this asymmetrical association between the two variables.

The AOP measure compares two scenarios for predicting the class label for a randomly chosen instance: (1) knowing only the class-distribution in the training data and (2) knowing both the class distribution and the value of a feature *F*. The AOP measure is calculated using the probability of error in both cases, and increases in value as the probability of error in scenario 2 reduces. The AOP score is calculated as follows.


$$E_{1}=1-max((p_{00}+p_{10}),(p_{01}+p_{11}))$$

$$E_{2}=1-\max(p_{00},p_{01})-\max(p_{10},p_{11})$$

$$\text{Asymmetric}\;\text{Optimal}\;\text{Prediction}=\frac{\left(E_{1}-E_{2}\right)}{E_{1}}$$

where p_00_ = n_00_/n, p_10_ = n_10_/n, p_01_ = n_01_/n, p_11_ = n_11_/n; n_00_, n_10_, n_01_ and n_11_ are as defined in Figure 2, *n* is the total number of training instances, *E_1_* is the probability of a prediction error when an instance is predicted to have the most frequent class label among all training instances (i.e. the class prediction ignores the feature value), and *E_2_* is the probability of a prediction error when an instance is predicted to have the most frequent class label among the training instances which have the same feature value (1 or 0) as the current instance (i.e. the class prediction is based on the feature value).

### Filter ensembles

As our datasets have imbalanced class distributions, the majority-class instances can skew the feature score values computed by a filter. One simple way to mitigate this would be to undersample the majority-class instances in the training set before calculating the filter scores, so that the data used by the filter is balanced [66]. However, this would cause most majority class instances to be completely ignored during the FS process.

Therefore, we applied instead a more robust way to calculate filter scores that also addresses the class imbalance issue, named Filter Ensembles. This strategy consists of using an ensemble of filters with bootstrap samples, combining their scores to get a final score value for each feature, which is calculated using only balanced datasets (i.e., each bootstrap sample has its majority class instances undersampled to a 1:1 ratio of positive- and negative-class instances). A similar strategy was used by [67] in the context of gene-gene interaction, and by [68] in the context of biomarker identification.

In our experiments we used an ensemble of 50 filters, but this number can be adjusted based on the available computational resources and the class distribution of the original dataset (more imbalanced datasets might need more filters). Each of the 50 balanced datasets is fed into a filter method to calculate the features’ scores, and the final score of a feature with that filter is the median value over these scores. This strategy is computationally expensive, but it can be implemented as a parallel algorithm, making use of multi-thread and multi-core architectures to reduce running time, as the scores for each of the 50 filters can be calculated simultaneously.

We performed a preliminary set of experiments comparing using the proposed Filter Ensembles strategy to not doing so (i.e., running a single filter using the full, unbalanced dataset), and concluded that the strategy led to classifiers with better predictive accuracy in general, and was therefore worthwhile. The discussion for this set of experiments is out of the scope of this paper, but the result tables detailing them are available in the Supplementary Material (Supplementary Tables 1–8).

### Auto-K: automatically selecting the number of top-ranked features to be kept

Generally, the user of a filter method needs to manually choose the number of top-ranked features to be selected by the method, called *k*. Naturally, this choice can significantly impact the performance of a classifier trained with the selected features. In order to make our filter methods more adaptable, and reduce the impact of subjective user choices of *k* in the predictive performance of classifiers, we used an automated process for selecting the best *k* value for a dataset out of a set of candidate k values, which we named Auto-K.

The candidate values of *k* were defined based on the numbers of features in the original datasets, as follows. For the Interactors and GO terms datasets, which have the two largest numbers of features, we set the candidate *k* values as 250, 500, 750 and 1000. For the Phenotypes datasets, which have relatively smaller numbers of features, we set the candidate *k* values as 100, 200, 300 and 400. Finally, for the GenAge/GenDR datasets, which have the smallest numbers of features, we set the candidate *k* values as 50, 100, 150 and 200 for the dataset version-1 and as 50, 100 for the dataset version-2.

The Auto-K selection works as follows. For each of the folds of the 10-fold external cross-validation, an internal 5-fold cross-validation is used to train RF classifiers using each candidate *k* value, and the value that results in models with the highest median AUC is chosen. Note that only the training data is used by the Auto-K process, as the test data cannot be accessed prior to evaluating the final classifier.

### The Auto-Filter method for feature selection

As the performance of a filter method depends largely on the data distribution, making an automated data-driven choice of the best filter method for each dataset intuitively should lead to better predictive performance, compared to making a fixed choice regardless of the data.

Thus, we implemented another filter approach named the Auto-filter approach. In addition to automatically selecting the best *k* value for the number of top-ranked features to be kept in the dataset, the Auto-Filter approach also selects the best candidate filter method out of the 5 candidate filters discussed in Section 4.2 or out of their filter ensemble counterparts (Section 4.3). This automated filter-method selection is based on an internal 5-fold cross-validation process applied to the training set, where it trains RF classifiers using each of the possible combinations of a filter (or filter ensemble) method and a *k* value.

The median AUC (Area Under the ROC curve) of the classifiers over the 5 folds of the internal cross-validation is used to select the best filter or filter ensemble method (the one with the highest median AUC), with the AUC variance as a tie-breaking criterion (the lower the variance, the better). As the Auto-Filter procedure is used inside an external cross-validation process, it will be run once for each fold in that external process, using each training dataset. Note that different candidate filter or filter ensembles and *k* values might be selected across the different folds of the external cross-validation. The pseudocode in Algorithm 1 represents the Auto-Filter procedure used in each fold of the external cross-validation.

**Algorithm 1:** The Auto-Filter procedure. It receives a set of candidate filter methods *S*, a set of candidate *k* values *K*, the training dataset of the external cross-validation, a classification algorithm (in this work, random forest) to be used in the internal comparisons of the candidate filters, and a target predictive performance metric (in this work, the AUC). It returns the candidate filter and *k* value combination with the largest average score. Note that in this pseudocode the term ‘filter method’ is being used in a generic way, it can denote either a single filter method or its counterpart filter ensemble.

1: **function** Auto-Filter(S, K, training_set, classifier, perf_metric) 2: candidate_filters = [ ] 3: internal_CV = createStratifiedCrossValidationSets(training_set,5) 4: **For each** filter **in** S: 5: **For each** k **in** K: 6: **For each** estimation_set, validation_set **in** internal_CV: 7: filter.calculateFeatureScores(estimation_set) 8: estimation_set.applyFilter(filter, k) 9: validation_set.applyFilter(filter, k) 10: c = trainClassifier(estimation_set, classifier) 11: score = c.Evaluate(validation_set, perf metric) 11: score_array.add(score) 12: candidate_filters.append([filter, k, score_array]) 13: **return** selectBestFilter(candidate_filters)

The Auto-Filter approach is flexible, as the user may select any number of candidate filters (with or without using filter ensembles) and *k* values, as well as the classification algorithm and performance metric used in its internal method for selecting the best candidate filter method (e.g., it might be more relevant to optimise the filter choice using the F-Score or the AUC metric, depending on the characteristics of the project). However, the Auto-Filter approach has the disadvantage of being computationally costly, as it requires many runs of each candidate filter and the classification algorithm. This disadvantage can be alleviated by a parallel implementation, as most parts of the process (i.e., calculating the scores of each candidate filter, the folds in the internal cross-validation, and the external cross-validation process) are independent.

### Experimental setup

In order to test the feature selection approaches described in this paper, we ran experiments comparing Random Forest (RF) classifiers trained using each of them. In all experiments we performed a 10-fold cross-validation process and report the median value of the well-known Area Under the Receiver Operating Characteristic curve (AUC) [69]. The RFs were trained with 500 trees and the number of features randomly sampled as candidate features for each node was $\sqrt{d}$ (rounded up to nearest integer, with 0.5 being rounded up), with *d* being the number of features in the current dataset.

As the datasets created for this research have a class-imbalance issue (about 3.4 majority class instances for every minority class instance), we trained our RFs using the Balanced Random Forest (BRF) method [31]. The BRF method draws a bootstrap sample of minority class instances for each tree in the forest, and randomly draws the same number of instances from the majority class instances, meaning the subset of instances used to generate each decision tree has a balanced ratio (1:1) of instances from each class.

### Data availability

The datasets used in the experiments and the program code for the feature selection methods will be made freely available on a repository at https://github.com/caioedurib/auto_filter.

## Supplementary Material

Supplementary Materials

Supplementary Tables
