# Supplementary material for: Predicting lifespan-extending chemical compounds for C. elegans with machine learning and biologically interpretable features
Source: Aging (Albany NY). 2023 Jul 13;15(13):6073–99. doi: 10.18632/aging.204866 (PMC10373959; doi:10.18632/aging.204866)
Supplement: Supplementary Materials [file aging-15-204866-s001.pdf]

SUPPORTING INFORMATION

SUPPLEMENTARY TABLES - COMPARING FEATURE SELECTION METHODS

Supplementary Table 1 through Supplementary Table 8 compare the median AUC values obtained by the random forest algorithm when using two different versions of each candidate filter method, with the highest value in each table highlighted in boldface. Supplementary Table 1 through Supplementary Table 4 refer to the "version-1" datasets and Supplementary Table 5 through Supplementary Table 8 refer to the "version-2" datasets. The difference between the version-1 and version-2 datasets is explained in Section 4.1 of the main paper.

Regarding the two versions of the candidate filter methods, the first version, named single filter, simply applies the standard filter method without using any ensemble or class balancing approach. Hence, it computes scores for the features using the dataset's original imbalanced form, where the majority class will usually have a larger impact on the score of a feature. The second version, named filter ensemble, addresses that issue by computing scores using an ensemble of balanced filters as proposed in Section 4.3 of the main paper. In order to determine whether the significant computational cost added by using the filter ensemble methods is worthwhile, we compared the single filter and filter ensemble methods in a set of experiments using the datasets prepared in this work, with two versions of a dataset for each of the four types of predictive feature. The last row of each table shows the AUC value for the baseline approach of simply training the classifier using the full set of features, without performing any feature selection in a pre-processing phrase. Note that, in all experiments, the value of *k* (the number of features selected by a filter or filter ensemble method) is automatically selected through the Auto-K process defined in Section 4.4 of the main paper.

Results comparing filter methods on the version-1 datasets

Discussion

As can be observed in Supplementary Tables 1–8, in 7 out of the 8 datasets, the best AUC value (highlighted in boldface in each table) was obtained by the filter ensemble approach. In addition, in total, over the 48 pairs of results comparing single filter vs filter ensemble methods (6 comparisons per table times 8 tables), the latter won in 40 (83%) of the cases. Hence, the filter ensemble approach clearly performed better than the single filter approach.

After deciding to apply the filter ensemble strategy, we then compared the filter ensembles' results to determine the best FS method (regarding predictive accuracy) out of our set of 6 candidate filter ensemble methods. The proposed Auto-Filter approach (described in Section 4.5) got the best median AUC results for two version-1 datasets, namely the Interactors_1 and the GOTerms_1 datasets. Other individual methods generated the best model in the other datasets; notably the Decision Stump ensemble filter won for 3 datasets.

SUPPLEMENTARY DATASETS

The datasets created for this study are available as tab-separated spreadsheets on our GitHub project (https://github.com/caioedurib/auto_filter), alongside a script to run the proposed Auto-Filter approach.

Supplementary Dataset 1.1. Protein interactors of DrugAge compounds (Version 1).

Supplementary Dataset 1.2. Protein interactors of DrugAge compounds (Version 2).

Supplementary Dataset 2.1. GO Term annotations for the Protein Interactors of DrugAge compounds (Version 1).

Supplementary Dataset 2.2. GO Term annotations for the Protein Interactors of DrugAge compounds (Version 2).

Supplementary Dataset 3.1. Physiology Phenotype annotations for the protein interactors of DrugAge compounds, based on WormBase Phenotype data (Version 1).

Supplementary Dataset 3.2. Physiology Phenotype annotations for the protein interactors of DrugAge compounds, based on WormBase Phenotype data (Version 2).

Supplementary Dataset 4.1. Ageing-related gene matches for the protein interactors of DrugAge compounds, based on the GenAge's and GenDR's lists of genes related to ageing and dietary restriction, respectively (Version 1).

Supplementary Dataset 4.2. Ageing-related gene matches for the protein interactors of DrugAge

compounds, based on the GenAge’s and GenDR’s lists of genes related to ageing and dietary restriction, respectively (Version 2).
