# Supplementary material for: Predicting lifespan-extending chemical compounds for C. elegans with machine learning and biologically interpretable features
Source: Aging (Albany NY). 2023 Jul 13;15(13):6073–99. doi: 10.18632/aging.204866 (PMC10373959; doi:10.18632/aging.204866)
Supplement: Supplementary Tables [file aging-15-204866-s002.pdf]

## SUPPLEMENTARY TABLES

**Supplementary Table 1. Median AUC values obtained by Random Forest using the single filter vs filter ensemble methods in a pre-processing phase – protein interactors dataset (version 1).**

| Interactors dataset | Single filter | Filter ensemble |
|---|---|---|
| Information Gain | 0.736 | 0.743 |
| Chi² | 0.730 | 0.738 |
| Decision Stump | 0.731 | 0.738 |
| Asymmetric Optimal Prediction | 0.724 | 0.742 |
| Log Odds Ratio | 0.725 | 0.713 |
| Auto-Filter | 0.759 | **0.801** |
| Baseline (no filter method) | 0.717 | |

**Supplementary Table 2. Median AUC values obtained by Random Forest using the single filter vs filter ensemble methods in a pre-processing phase – GO terms dataset (version 1).**

| GO terms dataset | Single filter | Filter ensemble |
|---|---|---|
| Information Gain | 0.753 | 0.744 |
| Chi² | 0.740 | 0.725 |
| Decision Stump | 0.719 | 0.734 |
| Asymmetric Optimal Prediction | 0.725 | 0.750 |
| Log Odds Ratio | 0.721 | 0.708 |
| Auto-Filter | 0.779 | **0.818** |
| Baseline (no filter method) | 0.767 | |

**Supplementary Table 3. Median AUC values obtained by Random Forest using the single filter vs filter ensemble methods in a pre-processing phase – physiology phenotypes dataset (version 1).**

| Phenotypes dataset | Single filter | Filter ensemble |
|---|---|---|
| Information Gain | 0.759 | 0.722 |
| Chi² | 0.711 | 0.729 |
| Decision Stump | 0.714 | **0.761** |
| Asymmetric Optimal Prediction | 0.755 | 0.741 |
| Log Odds Ratio | 0.724 | 0.706 |
| Auto-Filter | 0.719 | 0.728 |
| Baseline (no filter method) | 0.741 | |

**Supplementary Table 4. Median AUC values obtained by Random Forest using the single filter vs filter ensemble methods in a pre-processing phase – GenAge/GenDR dataset (version 1).**

| GenAge/GenDR dataset | Single filter | Filter ensemble |
|---|---|---|
| Information Gain | 0.709 | 0.727 |
| Chi² | 0.740 | 0.751 |
| Decision Stump | **0.757** | 0.742 |
| Asymmetric Optimal Prediction | 0.739 | 0.744 |
| Log Odds Ratio | 0.708 | 0.721 |
| DDFMS | 0.702 | 0.725 |
| Baseline | 0.683 | |

**Supplementary Table 5. Median AUC values obtained by Random Forest using the single filter vs filter ensemble methods in a pre-processing phase – protein interactors dataset (version 2).**

| Interactors dataset | Single filter | Filter ensemble |
|---|---|---|
| Information Gain | 0.695 | **0.752** |
| Chi² | 0.661 | 0.722 |
| Decision Stump | 0.689 | 0.704 |
| Asymmetric Optimal Prediction | 0.651 | 0.737 |
| Log Odds Ratio | 0.656 | 0.662 |
| Auto-Filter | 0.656 | 0.688 |
| Baseline (no filter method) | 0.747 | |

**Supplementary Table 6. Median AUC values obtained by Random Forest using the single filter vs filter ensemble methods in a pre-processing phase – GO terms dataset (version 2).**

| GO terms dataset | Single filter | Filter ensemble |
|---|---|---|
| Information Gain | 0.736 | 0.754 |
| Chi² | 0.692 | 0.73 |
| Decision Stump | 0.726 | **0.772** |
| Asymmetric Optimal Prediction | 0.69 | 0.768 |
| Log Odds Ratio | 0.667 | 0.713 |
| Auto-Filter | 0.669 | 0.708 |
| Baseline (no filter method) | 0.765 | |

**Supplementary Table 7. Median AUC values obtained by Random Forest using the single filter vs filter ensemble methods in a pre-processing phase – physiology phenotypes dataset (version 2).**

| Phenotypes dataset | Single filter | Filter ensemble |
|---|---|---|
| Information Gain | 0.694 | **0.718** |
| Chi² | 0.66 | 0.716 |
| Decision Stump | 0.695 | 0.701 |
| Asymmetric Optimal Prediction | 0.654 | 0.704 |
| Log Odds Ratio | 0.644 | 0.716 |
| Auto-Filter | 0.644 | 0.688 |
| Baseline (no filter method) | 0.715 | |

**Supplementary Table 8. Median AUC values obtained by Random Forest using the single filter vs filter ensemble methods in a pre-processing phase – GenAge/GenDR dataset (version 2).**

| GenAge/GenDR dataset | Single filter | Filter ensemble |
|---|---|---|
| Information Gain | 0.653 | 0.69 |
| Chi² | 0.638 | 0.685 |
| Decision Stump | 0.678 | 0.681 |
| Asymmetric Optimal Prediction | 0.64 | **0.72** |
| Log Odds Ratio | 0.617 | 0.69 |
| Auto-Filter | 0.628 | 0.692 |
| Baseline | 0.701 | |
